# Detection of Skin Cancer Based on Skin Lesion Images Using Deep Learning

**DOI:** 10.3390/healthcare10071183

**Published:** 2022-06-24

**Authors:** Walaa Gouda, Najm Us Sama, Ghada Al-Waakid, Mamoona Humayun, Noor Zaman Jhanjhi

**Affiliations:** 1 Department of Computer Engineering and Network, College of Computer and Information Sciences, Jouf University, Sakaka 72341, Al Jouf, Saudi Arabia; 2 Electrical Engineering Department, Faculty of Engineering at Shoubra, Benha University, Cairo 4272077, Egypt; 3 Faculty of Computer Science and Information Technology, Universiti Malaysia Sarawak, Kota Samarahan 94300, Malaysia; najmussama@gmail.com; 4 Department of Computer Science, College of Computer and Information Sciences, Jouf University, Sakaka 72341, Al Jouf, Saudi Arabia; gnalwakid@ju.edu.sa; 5 Department of Information Systems, College of Computer and Information Sciences, Jouf University, Sakaka 72341, Al Jouf, Saudi Arabia; 6 School of Computer Science and Engineering (SCE), Taylor’s University, Subang Jaya 47500, Malaysia; noorzaman.jhanjhi@taylors.edu.my

**Keywords:** deep learning, machine learning, convolutional neural network, ISIC 2018, skin lesion, computer vision

## Abstract

An increasing number of genetic and metabolic anomalies have been determined to lead to cancer, generally fatal. Cancerous cells may spread to any body part, where they can be life-threatening. Skin cancer is one of the most common types of cancer, and its frequency is increasing worldwide. The main subtypes of skin cancer are squamous and basal cell carcinomas, and melanoma, which is clinically aggressive and responsible for most deaths. Therefore, skin cancer screening is necessary. One of the best methods to accurately and swiftly identify skin cancer is using deep learning (DL). In this research, the deep learning method convolution neural network (CNN) was used to detect the two primary types of tumors, malignant and benign, using the ISIC2018 dataset. This dataset comprises 3533 skin lesions, including benign, malignant, nonmelanocytic, and melanocytic tumors. Using ESRGAN, the photos were first retouched and improved. The photos were augmented, normalized, and resized during the preprocessing step. Skin lesion photos could be classified using a CNN method based on an aggregate of results obtained after many repetitions. Then, multiple transfer learning models, such as Resnet50, InceptionV3, and Inception Resnet, were used for fine-tuning. In addition to experimenting with several models (the designed CNN, Resnet50, InceptionV3, and Inception Resnet), this study’s innovation and contribution are the use of ESRGAN as a preprocessing step. Our designed model showed results comparable to the pretrained model. Simulations using the ISIC 2018 skin lesion dataset showed that the suggested strategy was successful. An 83.2% accuracy rate was achieved by the CNN, in comparison to the Resnet50 (83.7%), InceptionV3 (85.8%), and Inception Resnet (84%) models.

## 1. Introduction

The uncontrollable development of tissues in a specific body area is known as cancer [1]. One of the most quickly spreading diseases in the world looks to be skin cancer. Skin cancer is a disease in which abnormal skin cells develop out of control [2]. In order to determine potential cancer therapies, early detection and accurate diagnosis are essential. Melanoma, the deadliest form of skin cancer, is responsible for most skin cancer-related deaths in developed countries. The major skin cancer types comprise basal cell carcinoma [3], squamous cell carcinoma [4], Merkel cell cancer [5], dermatofibroma [6], vascular lesion [7], and benign keratosis [8].

**Figure 1:**
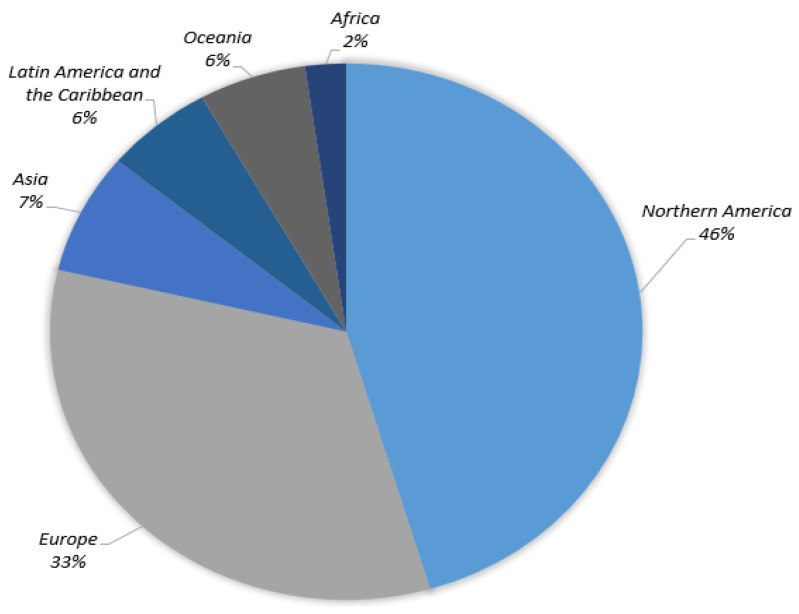
Skin cancer cases globally (22 March 2022) [1].

In order to diagnose abnormalities in various regions of the body, such as skin cancer [9], breast cancer [10], brain tumors [11], lung cancer [12], and stomach cancer [13], diagnostic imaging assessment plays an important part. According to the GLOBOCAN survey, there will be 19.2 million new cancer diagnoses and 9.9 million cancer deaths in 2020. Lung cancer is the leading cause of death (18.2%), followed by colorectal cancer (9.5%), liver cancer (8.4%), stomach cancer (7.8%), breast cancer (6.9%), esophageal cancer (5.5%), and pancreatic cancer (4.7%). The GLOBOCAN survey also points out more than half of cancer deaths occur in Asia, along with about 20% of cancer deaths occurring in Europe. Furthermore, the areas most affected by skin cancer around the globe are shown in Figure 1, with North America accounting for about half of the total.

To ensure better prognosis and death rates, early skin cancer identification is crucial, yet solid tumor detection typically relies mostly on screening mammography with inadequate sensitivity, which is then validated by clinical specimens. Cancer screening and treatment reaction evaluations are usually not appropriate uses for this approach [2,3]. An increasing number of healthcare providers are using artificial intelligence (AI) for medical diagnostics to improve and accelerate the diagnosis decision-making procedure [4]. However, despite some current evidence of improvement in this domain, the accurate assessment and adequate reporting of predicted flaws have been entirely or partly ignored by currently available AI research for clinical diagnosis.

**Figure 2:**
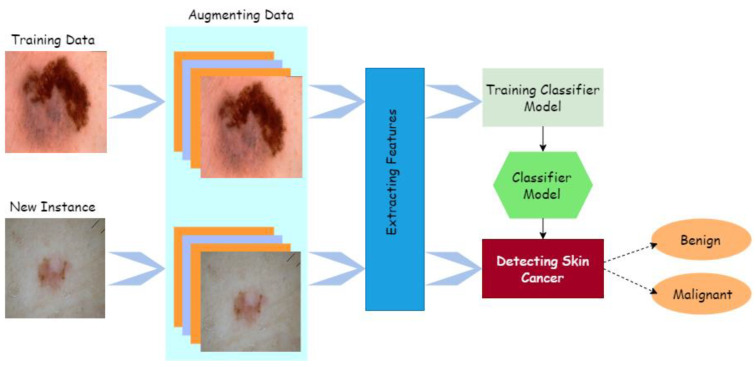
Process of cancer detection.

Computer-aided design (CAD) can quickly, reliably, and consistently diagnose various disorders. CAD also provides the option for advanced tumor disease detection and protection that is both precise and cost-effective. Human organ disorders are typically assessed using a variety of imaging technologies, including magnetic resonance imaging (MRI) [5], positron emission tomography (PET) [6], and X-rays [7]. Computed tomography (CT) [8,9], dermatoscopy image analysis, clinical screening, and other approaches were initially used to visually diagnose skin lesions. Dermatologists with little expertise have shown reduced accuracy in skin lesion diagnostics [10,11,12]. The methods for physicians to evaluate and analyze lesion images are time-consuming, complex, subjective, and error-prone. This is mainly because the images of skin lesions are so complicated. Unambiguous identification of lesion pixels is essential to performing image analysis, for evaluation and awareness of skin lesions. Using machine learning approaches in computer vision has led to a significant advance in computer-aided diagnostic and prediction systems for skin cancer detection [13]. Image preprocessing and classification of lesion images are some of the main processes used to outline the entire cancer detection and diagnosis, as described in Figure 2 [14].

The exponential growth in processing power has led to tremendous advancements in computer vision technologies, particularly in the development of deep learning models such as CNN. The earliest possible detection of skin cancer is now required. Skin cancer is the second most common cancer (after breast cancer) in women between the ages of 30 and 35, and the most common cancer in women between the ages of 25 and 29, according to Dr. Lee [15], who serves several young patients with skin cancer. Early identification of skin cancer using deep learning outperformed human specialists in many computer vision challenges [15,16], resulting in reduced death rates. It is possible to get outstanding and cutting-edge processing and classification accuracy by including efficient formulations into deep learning techniques [17,18,19].

In order to correctly diagnose early cancer signs from lesion images, this study proposes a crossbred DL model for cancer classification and prediction. Preprocessing and classification are key components of the system under consideration. During the preprocessing phase, the entire intensity of the image is improved to decrease the inconsistencies among photos. The image is additionally scaled and standardized to fit the training model’s scale during this procedure. Many different metrics were used to evaluate the suggested model in the comparison studies. These metrics included precision and recall metrics, the F1-score, and the area under the curve (AUC). The publicly available, large-scale ISIC 2018 dataset comprises a massive number of lesion images with diagnosed cancer. Pretrained networks such as Resnet50, InceptionV3, and Inception Resnet were employed for comparison. A training process with varying configurations of training strategies (e.g., validation patience and data augmentation) was employed to boost the recommended technique’s universal efficiency and prevent overfitting.

The remainder of this paper is broken down as follows: Section 2 summarizes existing investigations, Section 3 describes the methods used to build the cancer dataset and the recommended system’s design requirements, Section 4 offers the findings of the study, and Section 5 finishes with the conclusion and suggestions for further studies.

## 2. Related Work

Skin cancer is on the upswing, and this has been true for the last 10 years [20]. Because the skin is the body’s central part, it is reasonable to assume that skin cancer is the most frequent disease in humans. Timely detection of skin cancer is essential for successful therapy. Skin cancer indications can now be quickly and easily diagnosed using computer-based techniques. Multiple noninvasive methods have been proposed for assessing skin cancer signs.

The use of machine aid in the early diagnosis of cancer has opened up a new field of study and demonstrated the ability to eliminate limitations in the manual method. An overview of several relevant studies is presented here to better understand the topic of discussion and to create a vision of the current state of the art. Deep learning techniques have produced outstanding outcomes in several areas compared to other traditional machine learning methodologies. In the last few decades, deep learning has completely transformed the nature of machine learning. The artificial neural network is the most advanced branch of machine learning. The anatomy and operation of the human brain was the source of inspiration for this method [21].

Experts have examined and assessed the strength of the facts supporting the accuracy rate of computer-aided techniques [22]. ScienceDirect, SpringerLink, and IEEE databases were consulted. Skin lesion segmentation and classification approaches were analyzed, outlining the significant limitations. An enhanced melanoma skin cancer diagnosis technique was presented in [23]. An implantation manifold with nonlinear embeddings was used to create synthetic views of melanoma. Employing dermatoscopic scans from the publicly accessible PH^2^ dataset, the data augmentation approach was utilized to build a new collection of skin melanoma datasets. The SqueezeNet deep learning model was trained using the enhanced images. The experiments revealed that the accuracy of melanoma identification improved significantly (92.18). Extracting a skin melanoma (SM) region from a digital dermatoscopy image using the VGG-SegNet algorithm was suggested in [24]. Essential performance parameters were subsequently established after a comparison between the extracted segmented SM and the ground truth (GT). Employing the standard ISIC2016 database, the proposed scheme was evaluated and verified.

Scholars have combined human and artificial intelligence to classify skin cancer. A total of 112 German dermatologists and a CNN categorized 300 biopsy-verified skin lesions into five classifications. Using gradient boosting, the two separately obtained sets of diagnoses were joined to create a unified classifier. Man and machine obtained 82.95% multiclass accuracy [25]. The deep learning-based InSiNet technique detects benign and malignant tumors [26]. Under similar scenarios, the approach was evaluated on HAM10000 images (ISIC 2018), ISIC 2019, and ISIC 2020. Accordingly, the created InSiNet framework outperformed the other approaches, obtaining 94.59%, 91.89%, and 90.549% accuracy when using the ISIC 2018, ISIC 2019, and ISIC2020 datasets.

To categorize skin melanoma at an early stage, researchers offered a deep-learning-based methodology, including a region-based convolutional neural network (RCNN) and fuzzy k-means clustering (FKM) [27]. The suggested technique was put to the test using a variety of clinical photos in order to aid dermatologists in the early detection of this life-threatening condition. The ISIC-2017, PH2, and ISBI-2016 datasets were used to assess the provided methodology’s effectiveness. The findings revealed that it outperformed current state-of-the-art methodologies with an average accuracy of 95.40%, 93.1%, and 95.6%.

DL models such as convolutional neural networks (CNNs) have proven themselves superior to more traditional methods in various fields, especially image and feature recognition [28]. Moreover, they have been effectively applied in the medical profession, with phenomenal results and outstanding performance in a variety of challenging situations. Doctors and professionals now have access to a variety of DL-based medical imaging systems to aid in cancer prognosis, treatment, and follow-up assessments.

The Lesion-classifier, relying on pixel-by-pixel classification findings, was presented to categorize skin lesions into melanoma and non-melanoma cases. Skin lesion datasets ISBI2017 and PH2 were used in the investigation to verify efficacy. The experiments showed that the suggested technique had an accuracy rate of 95% on the ISIC 2017 and PH2 datasets [29].

In recent years, various deep learning algorithms have been applied to classify skin cancer, as outlined in Table 1, as well as other existing studies such as [30,31]. Table 1 presents the various methods for predicting cancer.

Timely screening and prediction have been found to enhance the probability of proper medication and reduce mortality. However, most of these studies focused solely on applying DL models to actual images rather than preprocessed images, limiting the ultimate classification network’s ability to adapt. By altering the framework of pretrained systems via the addition of multiple layers, the present work builds a lightweight skin cancer diagnosis method in order to achieve a higher level of confidence.

## 3. Proposed System

A CNN model using images from the image data store is presented schematically to generate discriminative and relevant attribute interpretations for the cancer detection technique, as shown in Algorithm 1. To begin, a basic explanation of the used dataset is provided. Moreover, the details of the implementation of proposed model, including preprocessing techniques and the basic architecture, are presented.
**Algorithm 1:** Processes in the mechanism suggestedLet Ƀ = lesion image, aug = augmentation, ppr = preprocessing, ig = image, ig€ = image enhancement algorithm (ESRGAN), rt = rotation, sc = scaling, rl = reflection, and sh = shifting method Input: {Lesion image Ƀ} Output: {confusion matrix, accuracy, precision, ROC, F1, AUC, recall} Step 1: Browse(Ƀ) Step 2: Implement (ppr (ig)) 2.1. Operate (ig€)_ 2.2. aug(ig) w.r.t. rt, sc, rl, sh   2.2.1. perform rt   2.2.2. perform sc   2.2.3. perform rl   2.2.4. perform sh 2.3. Resize (ig)/224*24*3 2.4 Normalize pixelvalue (ig)/interval [0,1] Step 2: Split (dataset)/training, testing, and validating Step 3: Train CNN model Step 4: Train pretrained models (Resnet, Inception, Inception Resnet)   4.1 Fine-tune model parameters (freeze layers, learning rate, epochs, batch size) Step 5: Compute VPM (confusion matrix, accuracy, precision, ROC, F1, AUC, recall) Step 6: Evaluation (existing work)

### 3.1. ISIC 2018 Image Dataset

**Figure 3:**
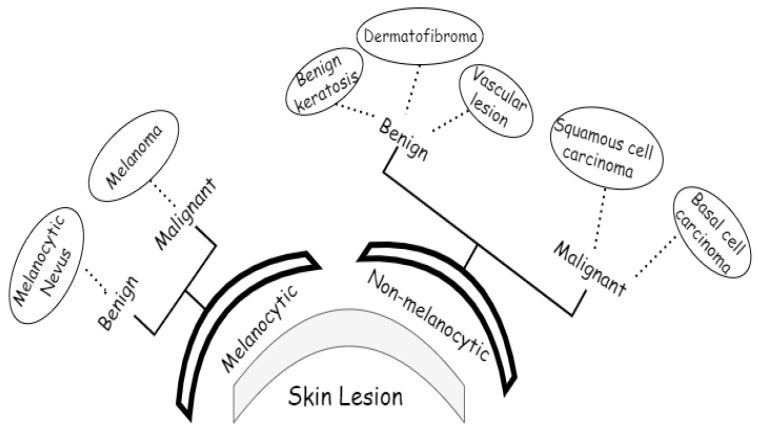
Classes of ISIC2018 dataset.

Data are at the core of DL, representing what these learning techniques run on. Cancer is a unique disease, and there have already been many datasets published. We used lesion images from publicly accessible image databases of identified affected individuals. The ISIC 2018 dataset was utilized for training the proposed approach, which contained 10,015 training and 1512 test images for a total of 11,527 images [30]. ISIC 2018 provided the ground-truth data only for the training set, consisting of seven classes, melanoma, melanocytic nevus, basal cell carcinoma, squamous cell carcinoma, vascular lesions, dermatofibroma, and benign keratosis, as shown in Figure 3.

**Figure 4:**
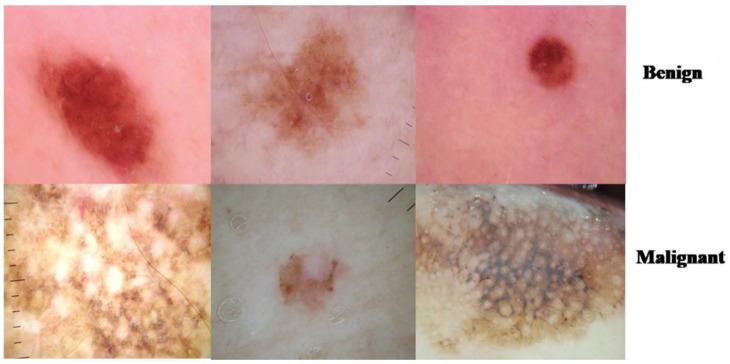
Lesion images from ISIC2018 dataset.

We applied the proposed CNN model to the ISIC 2018 skin lesion classification challenge test set; our data store consisted of 3533 lesion scans where 1760 of them are benign and 1773 are malignant, and we tested the proposed system using a total of 960 images consisting of 360 benign and 300 malignant cases. The lesion images were acquired from an openly accessible data repository ISIC 2018 [31]. For evaluation, the authors obtained radiological scans from many legitimate databases of cancer incidences; images from this source are used in most cancer diagnostics. The database, which is updated regularly, offers a free library of cancer cases and lesion images. The Kaggle list “Lesion Images” was used to collect lesion images; 3533 images from these sources are included in the ISIC2018 collection [41]. Figure 4 shows various lesion image examples from the ISIC2018 dataset, demonstrating the collection’s diversity of patient situations. It was decided to build ISIC2018 because the library is openly available and openly available to the academic communities and the public society.

### 3.2. Image Preprocessing

This process involved data augmentation, image improvement using (ESRGAN), image resizing, and normalization.

#### 3.2.1. ESRGAN

Approaches such as super-resolution generative adversarial network enhanced SRGAN [42] can help improve the detection of skin lesions. The enhanced edition of the super-resolution GAN (Ledig et al.) [43] uses a resilient-in-residual block instead of a basic residual network or a simple convolution trunk when it comes to microscopic-level gradients. Additionally, the model does not have a batch normalization layer for smoothing down the image. Accordingly, the sharp edges of the image artefacts can be better approximated in the images produced by ESRGAN. When determining if an image is real or false, ESRGAN employs a relativistic discriminator https://arxiv.org/pdf/1807.00734.pdf (accesed on 10 April 2022). This method yields more accurate results. Perceptual differences between the actual and false images are combined with the relativistic average loss and pixelwise absolute difference between the real and fake images as the loss function during adversarial training. A two-phase training scheme is used to sharpen the generator’s skills. This reduces the pixelwise L1 distance between the input and target high-resolution image to avoid local minima when beginning with complete randomization in the first phase of the algorithm.

In the second stage, the goal is to refine and improve the reconstructed images of the smallest artefacts. The final trained model is interpolated between the L1 loss and the adversarially trained models for a photorealistic reconstruction.

**Figure 5:**
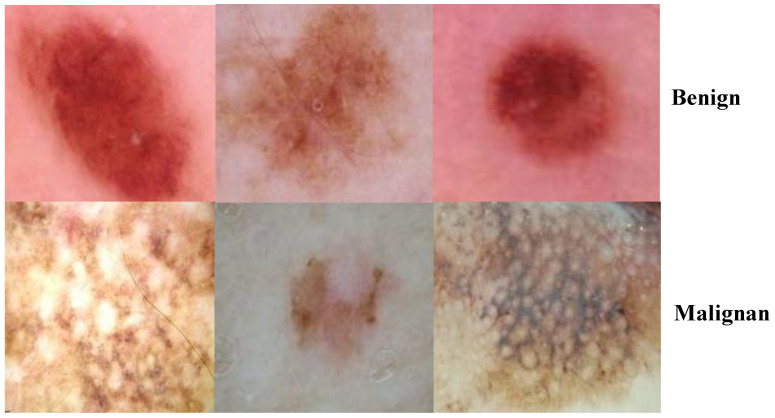
Images after the enhancement process.

A discriminator network was trained to distinguish between super-resolved images and actual photo images. By rearranging the lightness elements in the source image’s histogram, an evolutionary contrast enhancement algorithm was used to strengthen the lesion picture’s minutiae, textures, and poor contrast. As a result, this method enhanced the appearance of borders and arcs in each section of the picture, as shown in Figure 5, while simultaneously increasing the image’s contrast level.

#### 3.2.2. Augmentation

For each image in the dataset, upgraded images with associated masks including rotation, reflection, shifting, brightness, and resizing were produced. Detection and assessment are restricted by the poor quality of raw lesion images generated by electronic detectors. There were a total of 1440 benign and 1197 malignant training images. After conducting augmentation, there were a total of 1760 benign and 1773 malignant images. The imbalanced distribution of classes was addressed by performing oversampling on the malignant images.

**Figure 6:**
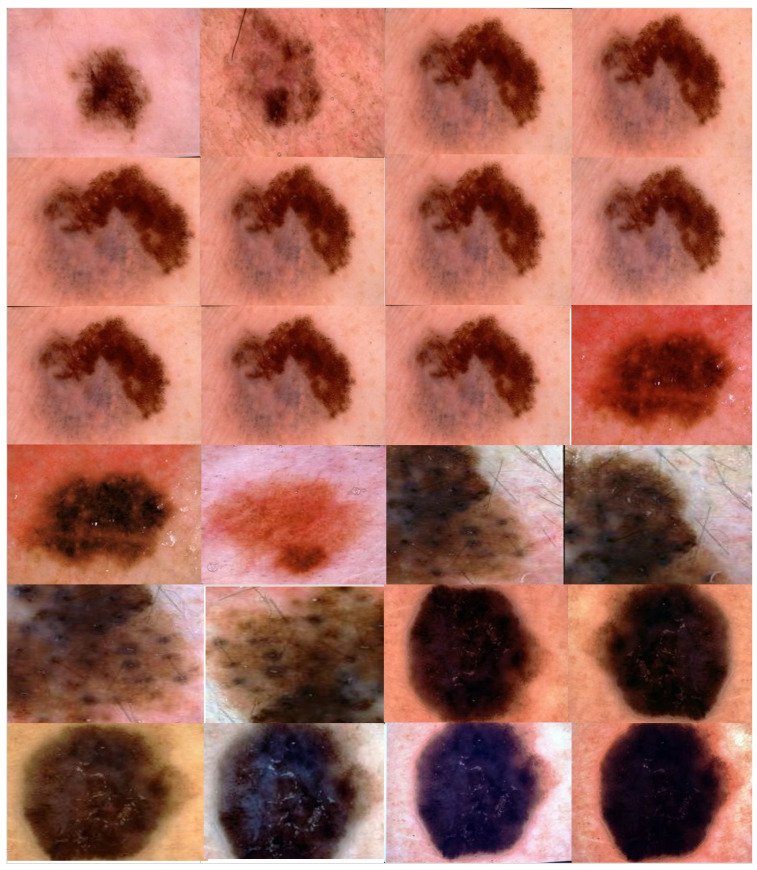
Output of the proposed image augmentation process.

To avoid biased prediction consequences, the ISIC2018 dataset was split into three mutually distinct sets (training, validation, and evaluation sets) to address the overfitting issue caused by the short number of training photographs. The output of the image augmentation process after applying different augmentation parameters is shown in Figure 6.

#### 3.2.3. Data Preparation

Image acquisition factors can vary due to the fact that certain photos in the dataset have low pixel dimensions, and all images should be resized. As a result, the image’s luminance and size can change dramatically. Each acquisition tool has its own unique set of criteria; hence, the lesion image dataset is likely to contain a variety of images. In order to verify that the data were consistent and free of noise, the pixel strength of all images was standardized within the interval [−1, 1]. Normalization computed using Equation (1) ensured that the model was less susceptible to minor weight changes, facilitating its improvement. Below, *I_norm_*, *Min_I_*, and *Max_I_* represent image, normalize, minimum, and maximum, respectively.

(1) $$I_{norm}=\left(I-Min_{I}\right)\left(\frac{2}{Max_{I}-Min_{I}}\right)-1$$

### 3.3. Proposed CNN for ISIC2018 Detection

**Figure 7:**
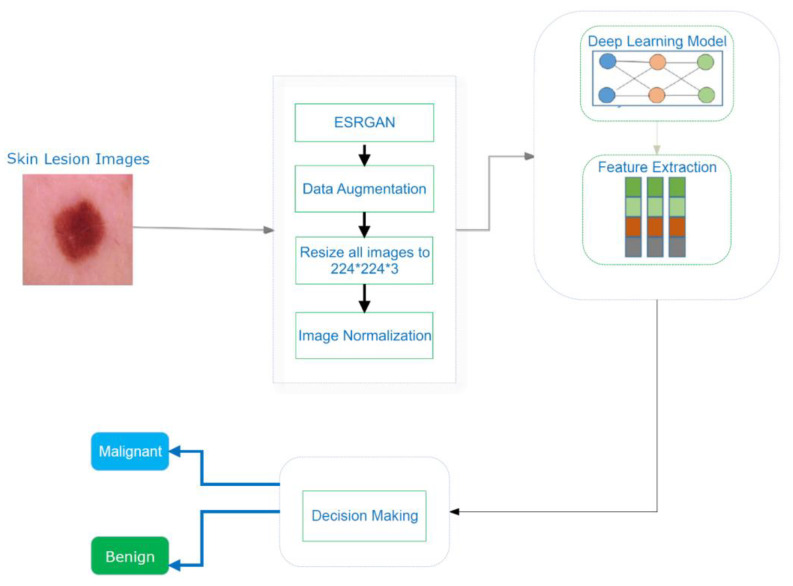
An illustration of the skin cancer detection technique.

Due to the enormous number of hyperparameters and structures that need to be accounted for, DL models face significant difficulty (e.g., learning rate, number of frozen layers, batch size, and number of epochs). Several hyperparameter values were tested to see how they affected the efficiency of the suggested systems. The proposed CNN model consisted of three layers, as shown in Figure 7. As depicted in Figure 7, the skin cancer detection system employed a transfer DL strategy to learn discriminative and informative feature representations from preprocessed images in the image dataset.

The presented system’s core architecture was built on three learning models: Resnet50, Inception, and Inception Resnet50.

#### 3.3.1. Resnet50

Resnet50is a 50-layer residual network [44]. Several difficulties emerged when scholars tried to apply the adage “the deeper the better” to deep learning methods. In comparison to networks having 20–30 layers, the deep network with 52 layers produced subpar outcomes, disproving the theory that “the deeper the network, the higher the network’s efficiency”. Resnet-50, a residual learning feature of the CNN model, was developed by experts. The residual unit is compensated for by using a conventional layer with a skip connection. Tying a layer’s incoming signal to a certain layer’s output using a skip connection is possible. The residual units allowed the training of a 152-layer model that was used to win the 2015 LSVRC2015 challenge. There is less of a learning curve because of its novel residual structure. A top five false-positive rate of <3.6% can be achieved using this machine.

#### 3.3.2. Inception V3

An essential feature of the Inception module is its capacity to perform multiresolution processing [45]. To capture characteristics in standard CNN models, kernels with distinct receptive areas are utilized in certain layers. In an inception model, on the other hand, many kernels with differing receptive fields are employed in tandem to retrieve features of various sizes. The Inception module’s outcome is created by stacking the parallel features that were extracted one on top of the other. The subsequent convolutional layer of the CNN uses the rich attribute maps produced by the Inception module’s merged result. Because of this, the Inception module’s effectiveness in medical imaging, specifically on lesion pictures, is exceptional [46].

#### 3.3.3. Inception Resnet

The Resnet50 and Inception frameworks were combined into one model to classify hyperspectral images. More than one million photos from the ImageNet collection were used to train the Inception ResnetV2 convolutional neural network. In total, there are 164 layers in this network, and it is capable of classifying photos into 1000 different object categories. Consequently, the network has amassed a diverse set of feature descriptions. The network accepts a 299-by-299-pixel picture as an input and gives a set of classifiers.

## 4. Experimental Results

Experiments were conducted on the ISIC2018 dataset to illustrate the effectiveness of the suggested DL systems and to compare their findings to those of the current state of the art.

### 4.1. Parameter Setting and Experimental Evaluation Index

**Figure 8:**
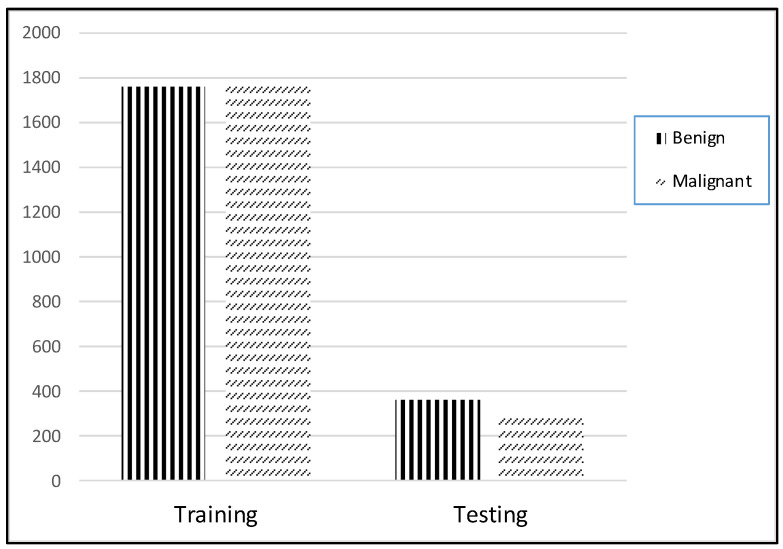
Distribution of dataset.

Simulations on the ISIC2018 dataset were carried out to illustrate the performance of the suggested DL systems and to compare their results to the current state of the art. On a linux desktop with a GPU RTX3060 and 8GB of RAM, the TensorFlow Keras program for the present scheme was tested. Training and testing sets were separated using a ratio of 80 to 20%, as shown in Figure 8. The training set contained 1760 benign and 1773 malignant images, while the testing set comprised 360 benign and 300 malignant images.

The suggested training set comprised an 80% randomized array of lesion images. All testing was conducted using this set. Then, 10% of the data were used for verification throughout the learning phase. The weight combinations with the greatest accuracy values were retained. On the ISIC2018 dataset, the Adam optimizer was used to pretrain the suggested architecture, which employs a learning rate technique that slows down learning when it becomes static for an extended period (i.e., validation patience). Furthermore, we implemented a batch rebalancing technique to improve the prevalence of infection forms during the batching process. The hyperparameters and their values used by the Adam optimizer for training are presented in Table 2.

### 4.2. Performance Assessment

This part of the study includes an in-depth explanation of the evaluation metrics utilized and their outcomes. Classifier accuracy (Acc) is the primarily used statistic for evaluating classification effectiveness. It is described as the number of instances (images) categorized accurately divided by the number of examples (images) in the dataset under analysis, as expressed in Equation (2). There are two used metrics generally used for evaluating the effectiveness of image categorization systems: precision (Pr) and recall (Rc). Precision is a measure of how many classified photos are correctly labeled compared to the total number of images, as expressed in Equation (3). Recall is the percentage of successfully categorized images in the database compared to the number of associated images, as expressed in Equation (4). The F-score is the harmonic mean of precision and recall; a greater value is an indicator of the system’s ability to forecast the future. The effectiveness of systems cannot be judged just on the basis of precision or recall. Equation (5) is the mathematical representation of the F-score (Fs).

(2) $$Acc=\frac{T^{p}+T^{n}}{T^{p}+T^{n}+F^{p\;}+F^{n}},$$

(3) $$Pr=\frac{T^{p}}{T^{p}+F^{p\;}},$$

(4) $$Rc=\frac{T^{p}}{T^{p}+F^{n\;}},$$

(5) $$Fs=2\times \left(\frac{Pr\times Rc}{Pr+Rc}\right),$$

where $T^{p}$ indicates a true positive, $T^{n}$ indicates a true negative, $F^{p\;}$ indicates a false positive, and $F^{n}$ indicates a false negative.

### 4.3. Performance of Different DCNN Models

Different DCNNs (CNN, Resnet50, Inception, and Inception Resnet) were implemented for training and testing tasks on the ISIC 2018 skin lesion classification challenge dataset. Using an 80–20 split between training and testing, the results are presented of various assessments on the ISIC2018 dataset for the suggested systems. This division was chosen to minimize the impact on execution time. CNN, Resnet50, Inception, and Inception Resnet models were trained for 50 epochs employing 10% of the training set as a validation set, with a batch size ranging from 2 to 32, and learning rates varying from 1 × 10^4^ to 1 × 10^6^. Moreover, fine-tuning was performed regarding Resnet50, Inception, and Inception Resnet by freezing different numbers of layers to achieve the best accuracy. In order to train the models using similar parameters (runs 1–3, Table 3, Table 4, Table 5 and Table 6), we used several runs (three runs for the similar parameters) to construct the model ensemble. The accuracy fluctuated from run to run since the weights were generated at random for each run; only the best run outcome was saved. Table 3, Table 4, Table 5 and Table 6 show the accuracy results for the proposed CNN. 

**Figure 9:**
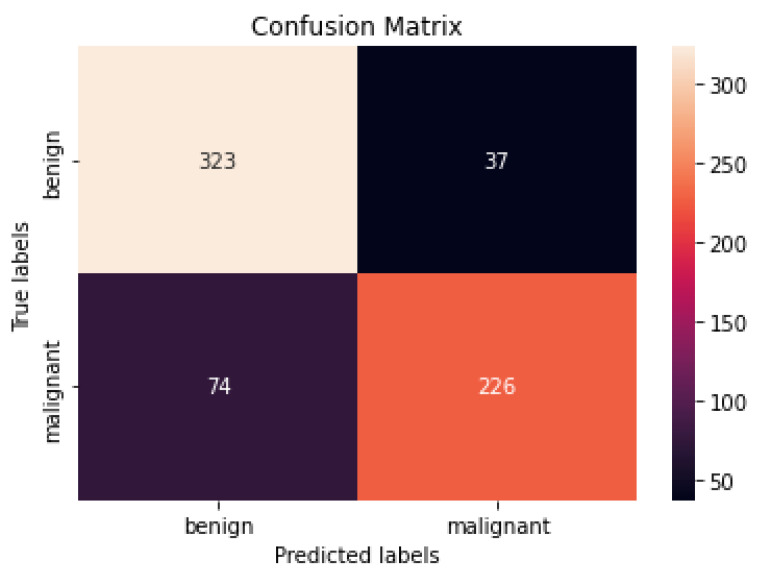
Best confusion matrix of CNN.

**Figure 10:**
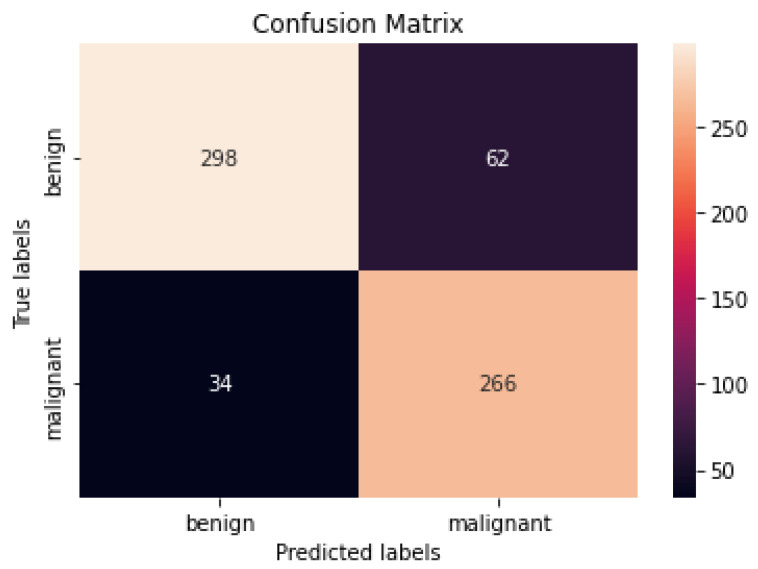
Best confusion matrix of InceptionV3.

Figure 9 and Figure 10 show the confusion matrices of the benign and malignant cancer findings using CNN, Resnet50, InceptionV3, and Inception Resnet. A total of 37 benign infected pictures were misinterpreted as malignant, while 74 malignant images were inaccurately classified as benign when using the CNN, in contrast to 62 benign images misclassified as malignant and 34 malignant images misinterpreted as benign when using InceptionV3.

**Figure 11:**
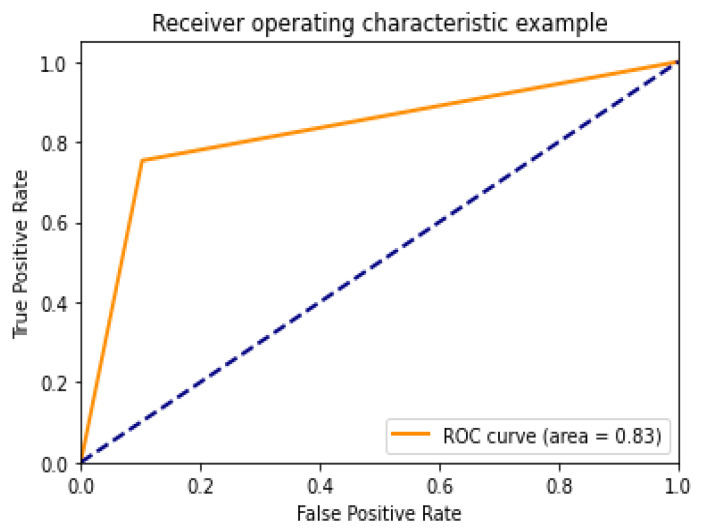
ROC curve for CNN model.

**Figure 12:**
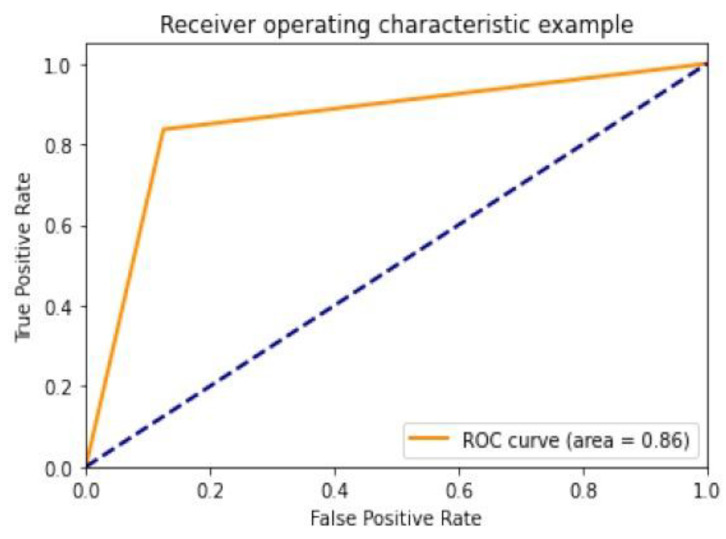
ROC curve for InceptionV3.

Diagnostic effectiveness was assessed using the AUC receiver operating characteristic curve (ROC), depicting the model’s categorization effectiveness as a function of two parameters: true positives and false positives. The AUC is calculated as the area under the ROC curve covered by small trapezoidal segments. As shown in Figure 11, we performed ROC analyses using a CNN model with an area of 0.83. The best-case ROC outcome for the suggested model after fine-tuning using InceptionV3 is shown in Figure 12.

It is now clear that the proposed strategy can be used in real-world settings to help radiologists diagnose cancer infection more correctly by utilizing lesion images, while simultaneously lowering their burden.

### 4.4. Comparison with Other Methods

A comparison of the suggested method’s efficacy to that of existing methods was performed to better demonstrate its viability. Table 7 shows that our strategy was superior to other networks in terms of performance. In the proposed approach, the Inception model had an overall accuracy rate of 85.7%, outperforming the existing models.

### 4.5. Discussion

According to our findings, none of the other approaches could match our level of precision. We attribute this to (i) ESRGAN’s overall resolution improvement, (ii) the fine-tuning to learn particular dataset aspects, and (iii) our use of numerous architectures, each with a different capacity to generalize and adapt to various data. The lack of unique medical image features meant that the transfer learning architectures could not achieve a higher level of classification accuracy. Despite being better at classifying natural pictures, Resnet50’s classification accuracy was lower than that of InceptionV3 when used on medical images. These findings suggest that shallower networks, such as that in InceptionV3, have more generalizable properties that may be used for a larger variety of imagery. On the other hand, deeper networks such as Resnet50 and Inception Resnet learn abstract characteristics that may be applied to any domain. Because the features of InceptionV3 are less semantically suited to natural pictures, they are more generalizable and adaptable when applied to medical images (compared to Resnet50 and Inception Resnet). Furthermore, fine-tuning the networks improved the accuracy of the four models. Compared to Resnet50 and Inception Resnet, InceptionV3’s accuracy increased the most. According to the results of this study, deep networks are more likely to acquire relevant features when fine-tuned on a smaller dataset than shallow networks. The confusion matrices and numerical data shown in Figure 9 and Figure 10 indicate that the suggested procedures were sufficient.

## 5. Conclusions and Future Work

By analyzing images of lesions on the skin, we developed a technique for quickly and accurately diagnosing both benign and malignant forms of cancer. The suggested system uses image enhancement approaches to boost the luminance of the lesion image and reduce noise. Resnet50, InceptionV3, and Resnet Inception were all trained on the upper edge of the preprocessed lesion medical images to prevent overfitting, as well as improve the overall competencies of the suggested DL methods. A lesion image dataset called the ISIC2018 dataset was used to test the proposed system’s performance. In the proposed approach, the Inception model had an overall accuracy rate of 85.7%, which is comparable to that of experienced dermatologists. In addition to experimenting with several models (designed CNN, Resnet50, InceptionV3, and Inception Resnet), this study’s innovation and contribution are the use of ESRGAN as a preprocessing step. Our designed model showed results comparable to the pretrained model. According to the comparative research, the proposed system outperformed current models. To establish the effectiveness of the suggested method, there is a need to conduct tests on a large, complex dataset that includes many cancer cases. It is possible that, in the future, we will employ Densenet, VGG, or AlexNet to analyze the cancer dataset.

## Figures and Tables

**Table 1:** Current methods, datasets, and results for skin cancer detection.

Recent Work	Data Size	Data Set	Techniques Used	Number of Classes
[25]	300	HAM10000	CNN with XGBoost	Five
[26]	1323	HAM10000	InSiNet	Two
[27]	1280	ISIC-2016	Region-based CNN (RCNN)	Two
	2000	ISIC-2017		
	200	PH^2^		
[29]	2000	ISBI2017	Deep convolutional encoder–decoder network (DCNN)	Two
[32]	48,373	DermNet, ISIC Archive, Dermofit image library	MobileNetV2	Two
[33]	7470	HAM10000	ResNet50	Seven
[34]	3753	ImageNet	ECOC SVM	Two
[35]	16,170	HAM10000	Anisotropic diffusion filtering	Two
[36]	1000	ISIC	SVM + RF	Eight
[37]	6705	HAM10000	DCNN	Two
[38]	279	ImageNet	DCNN VGG-16	Two
[39]	10,015	HAM10000	AlexNet	Seven
[40]	10,015	HAM10000	CNN	Seven

**Table 2:** Hyperparameters of Adam optimizer.

Parameter	Value
Batch size	2–32
Loss function	categorical cross-entropy
Momentum	0.95

**Table 3:** Average accuracy of CNN model using ISIC dataset (optimizer = Adam, learning rate = 1 × 10^−6^).

Batch Size	Ensemble Using Several Runs		
	Run 1	Run 2	Run 3
2	0.7818	0.7606	0.7011
4	0.7636	0.7833	0.7363
8	0.7363	0.75	0.7439
16	0.7939	0.7727	0.7636
32	0.7651	0.7363	0.7363

**Table 4:** Average accuracy of CNN model using ISIC dataset (optimizer = Adam, learning rate = 1 × 10^−5^).

Batch Size	Ensemble Using Several Runs		
	Run 1	Run 2	Run 3
2	0.8212	0.8196	0.8136
4	0.8121	0.8227	0.7924
8	0.8227	0.8227	0.8167
16	0.8000	0.7651	0.7985
32	0.8045	0.8136	0.8152

**Table 5:** Average accuracy of CNN model using ISIC dataset (optimizer = Adam, learning rate = 1 × 10^−4^).

Batch Size	Ensemble Using Several Runs		
	Run 1	Run 2	Run 3
2	0.8182	0.8000	0.8136
4	0.8318	0.8257	0.8121
8	0.8061	0.7909	0.8091
16	0.7879	0.7879	0.7985
32	0.7864	0.7969	0.7985

**Table 6:** Best accuracy after fine-tuning using several transfer learning models.

CNN	Resnet50	InceptionV3	Inception Resnet
0.8318	0.8364	0. 8576	0.8409

**Table 7:** Comparison with other methods.

Reference	Dataset	Model	Accuracy
[47]	ISIC2018	VGG19_2	76.6%
[48]	ISIC2016	VGGNet	78.6%
[49]	ISBI2017	AlexNet + VGGNet	79.9%
[50]	ISIC2017	U-Net	80.0%
[51]	2-ary, 3-ary, 9-ary	DenseNet	82%
[52]	HAM10000	AlexNet	84%
[53]	HAM10000	MobileNet	83.9%
Proposed	ISIC2018	CNN	83.1%
Proposed	ISIC2018	Resnet50	83.6%
Proposed	ISIC2018	Resnet50-Inception	84.1%
Proposed	ISIC2018	Inception V3	85.7%

## Data Availability

Data set is available at https://challenge.isic-archive.com/data/, 20 April 2022.

## References

[1] World Health Organization (2022). Global Health Observatory.

[2] Han H.S., Choi K.Y. (2021). Advances in nanomaterial-mediated photothermal cancer therapies: Toward clinical applications. Biomedicines.

[3] Fuzzell L.N., Perkins R.B., Christy S.M., Lake P.W., Vadaparampil S.T. (2021). Cervical cancer screening in the United States: Challenges and potential solutions for underscreened groups. Prev. Med..

[4] Ting D.S., Liu Y., Burlina P., Xu X., Bressler N.M., Wong T.Y. (2018). AI for medical imaging goes deep. Nat. Med..

[5] Wolf M., de Boer A., Sharma K., Boor P., Leiner T., Sunder-Plassmann G., Moser E., Caroli A., Jerome N.P. (2018). Magnetic resonance imaging T1-and T2-mapping to assess renal structure and function: A systematic review and statement paper. Nephrol. Dial. Transplant..

[6] Hooker J.M., Carson R.E. (2019). Human positron emission tomography neuroimaging. Annu. Rev. Biomed. Eng..

[7] Jaiswal A.K., Tiwari P., Kumar S., Gupta D., Khanna A., Rodrigues J.J. (2019). Identifying pneumonia in chest X-rays: A deep learning approach. Measurement.

[8] Morawitz J., Dietzel F., Ullrich T., Hoffmann O., Mohrmann S., Breuckmann K., Herrmann K., Buchbender C., Antoch G., Umultu L. (2022). Comparison of nodal staging between CT, MRI, and [18F]-FDG PET/MRI in patients with newly diagnosed breast cancer. Eur. J. Nucl. Med. Mol. Imaging.

[9] Jinzaki M., Yamada Y., Nagura T., Nakahara T., Yokoyama Y., Narita K., Ogihara N., Yamada M. (2020). Development of upright computed tomography with area detector for whole-body scans: Phantom study, efficacy on workflow, effect of gravity on human body, and potential clinical impact. Investig. Radiol..

[10] Celebi M.E., Codella N., Halpern A. (2019). Dermoscopy image analysis: Overview and future directions. IEEE J. Biomed. Health Inform..

[11] Barata C., Celebi M.E., Marques J.S. (2018). A survey of feature extraction in dermoscopy image analysis of skin cancer. IEEE J. Biomed. Health Inform..

[12] Adeyinka A.A., Viriri S. (2018). Skin lesion images segmentation: A survey of the state-of-the-art. Proceedings of the International Conference on Mining Intelligence and Knowledge Exploration.

[13] Al-Masni M.A., Al-Antari M.A., Choi M.-T., Han S.-M., Kim T.-S. (2018). Skin lesion segmentation in dermoscopy images via deep full resolution convolutional networks. Comput. Methods Programs Biomed..

[14] Ünver H.M., Ayan E. (2019). Skin lesion segmentation in dermoscopic images with combination of YOLO and grabcut algorithm. Diagnostics.

[15] Hu Z., Tang J., Wang Z., Zhang K., Zhang L., Sun Q. (2018). Deep learning for image-based cancer detection and Diagnosis—A survey. Pattern Recognit..

[16] Fujisawa Y., Otomo Y., Ogata Y., Nakamura Y., Okiyama N., Ohara K., Fujimoto M., Fujita R., Ishitsuka Y., Watanabe R. (2019). Deep-learning-based, computer-aided classifier developed with a small dataset of clinical images surpasses board-certified dermatologists in skin tumour diagnosis. Br. J. Dermatol..

[17] Adegun A., Viriri S. (2021). Deep learning techniques for skin lesion analysis and melanoma cancer detection: A survey of state-of-the-art. Artif. Intell. Rev..

[18] Iqbal S., Siddiqui G.F., Rehman A., Hussain L., Saba T., Tariq U., Abbasi A.A. (2021). Prostate cancer detection using deep learning and traditional techniques. IEEE Access.

[19] Dildar M., Akram S., Mahmood A.R., Mahnashi M.H., Alsaiari S.A., Irfan M., Khan H.U., Saeed A.H.M., Ramzan M., Alraddadi M.O. (2021). Skin cancer detection: A review using deep learning techniques. Int. J. Environ. Res. Public Health.

[20] Vaishnavi K., Ramadas M.A., Chanalya N., Manoj A., Nair J.J. Deep learning approaches for detection of COVID-19 using chest X-ray images. Proceedings of the 2021 Fourth International Conference on Electrical, Computer and Communication Technologies (ICECCT).

[21] Duc N.T., Lee Y.-M., Park J.H., Lee B. (2022). An ensemble deep learning for automatic prediction of papillary thyroid carcinoma using fine needle aspiration cytology. Expert Syst. Appl..

[22] Kassem M.A., Hosny K.M., Damaševičius R., Eltoukhy M.M. (2021). Machine learning and deep learning methods for skin lesion classification and Diagnosis: A systematic review. Diagnostics.

[23] Abayomi-Alli O.O., Damasevicius R., Misra S., Maskeliunas R., Abayomi-Alli A. (2021). Malignant skin melanoma detection using image augmentation by oversamplingin nonlinear lower-dimensional embedding manifold. Turk. J. Electr. Eng. Comput. Sci..

[24] Kadry S., Taniar D., Damaševičius R., Rajinikanth V., Lawal I.A. Extraction of abnormal skin lesion from dermoscopy image using VGG-SegNet. Proceedings of the 2021 Seventh International Conference on Bio Signals, Images, and Instrumentation (ICBSII).

[25] Hekler A., Utikal J.S., Enk A.H., Hauschild A., Weichenthal M., Maron R.C., Berking C., Haferkamp S., Klode J., Schadendorf D. (2019). Superior skin cancer classification by the combination of human and artificial intelligence. Eur. J. Cancer.

[26] Reis H.C., Turk V., Khoshelham K., Kaya S. (2022). InSiNet: A deep convolutional approach to skin cancer detection and segmentation. Med. Biol. Eng. Comput..

[27] Nawaz M., Mehmood Z., Nazir T., Naqvi R.A., Rehman A., Iqbal M., Saba T. (2022). Skin cancer detection from dermoscopic images using deep learning and fuzzy k-means clustering. Microsc. Res. Tech..

[28] Humayun M., Sujatha R., Almuayqil S.N., Jhanjhi N.Z. (2022). A Transfer Learning Approach with a Convolutional Neural Network for the Classification of Lung Carcinoma. Healthcare.

[29] Adegun A.A., Viriri S. (2019). Deep learning-based system for automatic melanoma detection. IEEE Access.

[30] Codella N., Rotemberg V., Tschandl P., Celebi M.E., Dusza S., Gutman D., Helba B., Kalloo A., Liopyris K., Marchetti M. (2019). Skin lesion analysis toward melanoma detection 2018, a challenge hosted by the international skin imaging collaboration (isic). arXiv.

[31] Kinyanjui N.M., Odonga T., Cintas C., Codella N.C., Panda R., Sattigeri P., Varshney K.R. (2020). Fairness of classifiers across skin tones in dermatology. Proceedings of the International Conference on Medical Image Computing and Computer-Assisted Intervention.

[32] Ech-Cherif A., Misbhauddin M., Ech-Cherif M. Deep neural network based mobile dermoscopy application for triaging skin cancer detection. Proceedings of the 2019 2nd International Conference on Computer Applications & Information Security (ICCAIS).

[33] Le D.N., Le H.X., Ngo L.T., Ngo H.T. (2020). Transfer learning with class-weighted and focal loss function for automatic skin cancer classification. arXiv.

[34] Dorj U.-O., Lee K.-K., Choi J.-Y., Lee M. (2018). The skin cancer classification using deep convolutional neural network. Multimed. Tools Appl..

[35] Rahman M.M., Nasir M.K., Nur A., Khan S.I., Band S., Dehzangi I., Beheshti A., Rokny H.A. (2022). Hybrid Feature Fusion and Machine Learning Approaches for Melanoma Skin Cancer Detection.

[36] Murugan A., Nair S.A.H., Preethi A.A.P., Kumar K.S. (2021). Diagnosis of skin cancer using machine learning techniques. Microprocess. Microsyst..

[37] Ali M.S., Miah M.S., Haque J., Rahman M.M., Islam M.K. (2021). An enhanced technique of skin cancer classification using deep convolutional neural network with transfer learning models. Mach. Learn. Appl..

[38] Guan Q., Wang Y., Ping B., Li D., Du J., Qin Y., Lu H., Wan X., Xiang J. (2019). Deep convolutional neural network VGG-16 model for differential diagnosing of papillary thyroid carcinomas in cytological images: A pilot study. J. Cancer.

[39] Rajput G., Agrawal S., Raut G., Vishvakarma S.K. (2022). An accurate and noninvasive skin cancer screening based on imaging technique. Int. J. Imaging Syst. Technol..

[40] Kanani P., Padole M. (2019). Deep learning to detect skin cancer using google colab. Int. J. Eng. Adv. Technol. Regul. Issue.

[41] (2021). MNOWAK061. Skin Lesion Dataset. ISIC2018 Kaggle Repository. https://www.kaggle.com/datasets/mnowak061/isic2018-and-ph2-384x384-jpg.

[42] Takano N., Alaghband G. (2019). Srgan: Training dataset matters. arXiv.

[43] Ledig C., Theis L., Huszar F., Caballero J., Cunningham A., Acosta A., Aitken A., Tejani A., Totz J., Wang Z. Photo-realistic single image super-resolution using a generative adversarial network. Proceedings of the IEEE Conference on Computer Vision and Pattern Recognition.

[44] He K., Zhang X., Ren S., Sun J. Deep residual learning for image recognition. Proceedings of the IEEE Conference on Computer Vision and Pattern Recognition.

[45] Wang C., Chen D., Hao L., Liu X., Zeng Y., Chen J., Zhang G. (2019). Pulmonary image classification based on inception-v3 transfer learning model. IEEE Access.

[46] Szegedy C., Ioffe S., Vanhoucke V., Alemi A. (2018). Inception-v4, inception-ResNet and the impact of residual connections on learning. arXiv.

[47] Foahom Gouabou A.C., Damoiseaux J.-L., Monnier J., Iguernaissi R., Moudafi A., Merad D. (2021). Ensemble Method of Convolutional Neural Networks with Directed Acyclic Graph Using Dermoscopic Images: Melanoma Detection Application. Sensors.

[48] Lopez A.R., Giro-i-Nieto X., Burdick J., Marques O. Skin lesion classification from dermoscopic images using deep learning techniques. Proceedings of the 2017 13th IASTED International Conference on Biomedical Engineering (BioMed).

[49] Harangi B. (2018). Skin lesion classification with ensembles of deep convolutional neural networks. J. Biomed. Inform..

[50] Kim C.-I., Hwang S.-M., Park E.-B., Won C.-H., Lee J.-H. (2021). Computer-Aided Diagnosis Algorithm for Classification of Malignant Melanoma Using Deep Neural Networks. Sensors.

[51] Alnowami M. (2019). Very Deep Convolutional Networks for Skin Lesion Classification. J. King Abdulaziz Univ. Eng. Sci..

[52] Ameri A. (2020). A deep learning approach to skin cancer detection in dermoscopy images. J. Biomed. Phys. Eng..

[53] Sae-Lim W., Wettayaprasit W., Aiyarak P. Convolutional neural networks using MobileNet for skin lesion classification. Proceedings of the 2019 16th International Joint Conference on Computer Science and Software Engineering (JCSSE).

